# Enhanced Sensitivity of Sub-THz Thermomechanical Bolometers Exploiting Vibrational Nonlinearity

**DOI:** 10.1021/acsphotonics.5c01613

**Published:** 2025-12-26

**Authors:** L. Alborghetti, B. Bertoni, L. Vicarelli, S. Zanotto, S. Roddaro, A. Tredicucci, M. Cautero, L. Gregorat, G. Cautero, M. Cojocari, G. Fedorov, P. Kuzhir, A. Pitanti

**Affiliations:** † Department of Physics, 9310 University of Pisa, Largo B. Pontecorvo 3, 56127 Pisa, Italy; ‡ NEST, CNR Istituto Nanoscienze, piazza San Silvestro 12, 56127 Pisa, Italy; ¶ Department of Physics, Università degli Studi di Trieste, Piazzale Europa 1, 34127 Trieste, Italy; § 18474 Elettra - Sincrotrone Trieste S.C.p.A., Strada Statale 14, km 163.5, 34149 Trieste, Italy; ∥ Department of Engineering and Architecture, Università degli Studi di Trieste, Via Alfonso Valerio 6/1, 34127 Trieste, Italy; ⊥ Department of Physics and Mathematics, Center of Photonics Sciences, 163043 University of Eastern Finland, Yliopistokatu 7, FI-80101 Joensuu, Finland

**Keywords:** terahertz detection, bolometer, room temperature, nonlinearity, micromechanical resonator, optomechanics

## Abstract

A common approach to detecting weak signals or minute quantities involves leveraging the localized spectral features of resonant modes, whose sharper lines (i.e., high Q-factors) enhance transduction sensitivity. However, maximizing the Q-factor often introduces technical challenges in fabrication and design. In this work, we propose an alternative strategy to achieve sharper spectral features by using interference and nonlinearity, all while maintaining a constant dissipation rate. Using far-infrared thermomechanical detectors as a test case, we demonstrate that signal transduction along an engineered response curve slope effectively reduces the detector’s noise equivalent power (NEP), achieving $∼30\;\mathrm{pW}/\sqrt{\mathrm{Hz}}$ NEP for electrical read-out, sub-THz detectors with an optimized absorbing layer.

## Graphical abstract

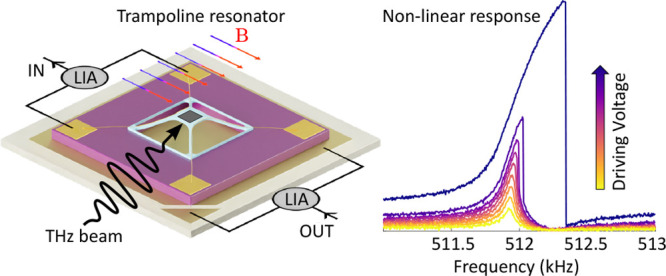

## Introduction

Transducer-based sensing, relying on the conversion of energy from one form (the measured quantity) to another (read-out signal), strongly benefits from spectrally sharp transfer functions, as large derivatives translate into large responsivities. The usual route for optimizing the transfer functions relies on devising resonant elements with low energy loss rates; this manifests into narrow line widths (large Q-factors) and leads to large sensitivities, which have been widely and successfully employed among the others in mass,^1^ aerostatic pressure,^2^ gas,^3^ temperature,^4^ polarization state^5^ and refractive index^6^ sensing.

Increasing the Q-factors of nano- and micrometric sized detectors, with footprints suitable for integration in electronic systems, represents a significant technological challenge, both in terms of design and fabrication. Noteworthy, an intense research has recently led to ultrahigh Q-factors (exceeding one billion) in micromechanical resonators oscillating at frequencies from hundreds of kHz to MHz, obtained through soft clamping and dissipation dilution design as well as careful fabrication.^7–10^ Although impressive, reaching consistently these values in commercially compatible processes remains a significant challenge: device imperfections or intrinsic physical effects often create bottlenecks, ultimately limiting the maximum sensitivity of commercial devices, which routinely have Q-factors ranging around 10^4^–10^5^.

A different, interesting route to obtain sharp features in the transfer functions relies on manipulating the resonant line shape, creating steeper edges while maintaining the same energy loss rate. To this end, Fano-induced asymmetry has been used for sensing enhancement, showing promising results for gas,^11–13^ refractive index^14,15^ and temperature^16^ sensing.

Improvements based on exploiting Fano lineshapes are still restricted by the limited manipulation of the Fano factor, which usually depends on the coupling between a broad and a narrow line width cavity system.^17^ Moreover, the maximum slope of a Fano response function is still connected to the resonator’s Q-factor. This further limit can be surpassed by taking advantage of intrinsic device nonlinearities, which can produce massive line width deformations (foldover effect^18^) or lead to multistability,^19^ with internal transitions between stable solutions leading to, in principle, “infinitely sharp” spectral features.

These concepts have been successfully implemented in several systems including superconductors^20^ and semiconductors^21,22^ single photon detectors. The same ideas have also found focused applications in micromechanical systems, including mass sensors monitoring the shift of nonlinear resonant frequencies,^23,24^ “threshold-based” sensors exploiting bifurcation phenomena for mass^25,26^ and gas^27,28^ limit detection, as well as more structured platforms exploiting exceptional points.^29^

In this paper, we show how mechanical nonlinearity enhanced transduction sensing can be employed to improve the characteristics of far-infrared light detectors based on thermo-mechanical bolometers (TMBs). TMBs have recently emerged as a powerful system which offers broadband detection at room-temperature, with single-pixel operation at video-rate and faster^30–34^ and the possibility of scaling up the system to focal plane arrays for multiplexed imaging applications.^35,36^ The best TMB devices have a noise-equivalent power (NEP) in a range from a few $\mathrm{pW}/\sqrt{\mathrm{Hz}}$ level, to some $\mathrm{nW}/\sqrt{\mathrm{Hz}}$ , in some cases outperforming commercially available technologies. A comprehensive and updated comparison of thermal THz detectors can be found in ref 37 (cf. Table 2 in ref 37) and in ref 38 (cf. Table 1 in ref 38), the latter specific to detector based on micromechanical resonators.

Our approach to TMBs makes use of silicon nitride (Si_3_N_4_) trampoline resonators, which have previously shown a NEP of about $100\;\mathrm{pW}/\sqrt{\mathrm{Hz}}$ at 20 Hz operating speed in a 1 × 1 mm sided membrane, illuminated with a 140 GHz source and optically read-out via self-mixing interferometry.^32^

Compared to our previous results, the devices here investigated have a reduced pixel size and employ specific layers for improved absorptance. In addition, addressing the TMB with metallic wires in a magnetic field, we switched to all-electrical probing via inductive read-out,^39^ which is better suited for integration and parallelization in large-scale arrays, with the trade-off of having a generically higher noise floor, primarily limited by Johnson white noise (not present in optical interferometric readout). While further mitigation strategies could exist to keep Johnson noise to minimal levels such as decreasing the contact resistance or cooling the device, we recognize that these could impact on the device mechanical quality and overall detection scheme architecture, making it less appealing for its possible use in field applications. Significant improvements in the noise characteristics could also be achieved by optimizing the read-out external setup, including amplifiers with minimal added noise and appropriate cabling and shielding configurations to minimize ground loops and possible undesired environmental signal pick-ups.

By applying mechanical nonlinearity enhanced transduction schemes, based on the Duffing effect,^40^ which emerges as a further correction of the system elastic constant due to intrinsic material and geometry-based nonlinearities,^41^ we achieve a NEP of about $30\;\mathrm{pW}/\sqrt{\mathrm{Hz}}$ , evaluated under an illuminating 140 GHz source and at room temperature. Combined with an operating speed of 20 Hz, our device characteristics and measurement technique challenge the state of the art for room-temperature bolometric detectors in the sub-THz range.

It is generally accepted that the high detector sensitivity comes at a price of reduced dynamic range. Extremely sensitive detectors are only used for extremely weak signals and cannot operate in a wide range of intensities due to saturation or even damage. In our case the degree of nonlinearity controlled through the amplitude of exciting voltage can be used to tune the detector from the regime of moderate sensitivity combined with high dynamic range (low excitation voltage, linear regime) to a state of small dynamic range and high sensitivity, required to detect very weak radiation.

## Methods

### Device Details

The devices are based on Si_3_N_4_ trampoline membrane resonators, which in the past decade have been successfully employed for classical sensing^42^ and quantum applications.^43,44^ The basic device geometry consists of single membranes made of a 300 nm thick stoichiometric silicon nitride, with a 100 × 100 μm central plate hanging on a 300 × 300 μm frame through four 12 μm wide tethers. This design has slightly longer and narrower tethers with respect to a previous report;^45^ this modification improves the device sensitivity by reducing the thermal coupling with the substrate at expenses of longer thermal relaxation time. All membranes have 50 nm thick Cr/Au metallic contacts with a width of approximately 10 μm running through the tethers, which are sufficient to ensure a strong electrical signal without adding excessive mass. These grant a practical all-electrical read-out and actuation,^39,46,47^ enabled by a 250 mT magnetic field induced by static Nd magnets and leveraging inductive reading or Lorentz force, respectively. A scanning electron micrograph of the investigated device is reported in Figure 1a along with a sketch outlining the read-out circuit.

**Figure 1:**
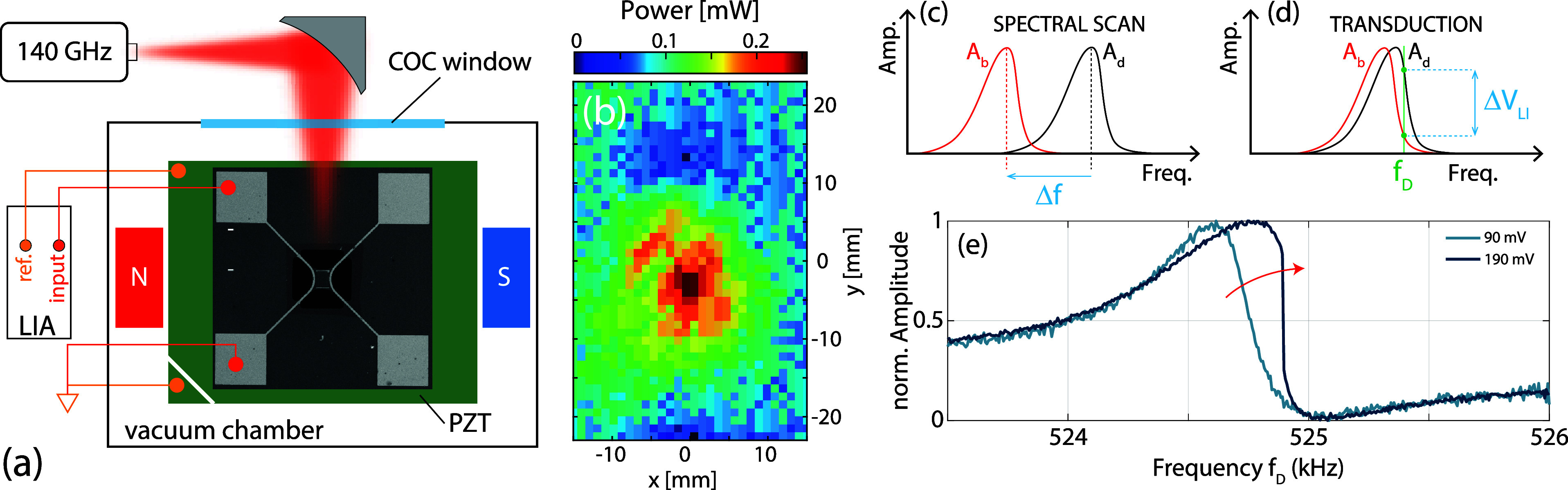
(a) Sketch of the experimental setup along with a SEM micrograph of one of the investigated TMB. The device sits on a piezoelectric actuator stack (PZT) in a vacuum chamber with optical access via a cyclic olefin copolymer (COC) window. Static magnets generate a planar magnetic field which is used for the coherent magneto-motive read-out demodulated by a lock-in amplifier (LIA) at the forcing mechanical drive. (b) Beam profiling of the 140 GHz source. Comparison of the Spectral Scan (c) and Transduction (d) operating modes. (e) Distortion of the resonance line shape of the trampoline resonator by increasing the driving amplitude and entering strong nonlinear motional regimes.

In order to enhance the device absorbance without degrading its mechanical properties, we introduced “ultra-light” bidimensional layers in the center of the membrane. We have explored the implementation of both metallic and carbon-based absorbers.^45^ The choice for metal was a Cr/Au bilayer with an overall thickness of about 8 nm. At such a small scale the metal layer will be a nonuniform film composed by adjacent grains, increasing its sheet resistance compared to its bulk metal value. This favored approaching the limit value of 188 Ω sheet resistance which would nominally gives perfect impedance matching and the theoretical 50% absorption limit for an isolated layer.^48,49^ However, while a ∼2 nm film would nominally achieve this condition more closely,^49^ such ultrathin layer lies below the percolation threshold and would require the introduction of additional materials (e.g., an oxidized copper layer) to obtain an homogeneous film, possibly hindering large-scale fabrication of detector arrays. The other avenue considered makes use of amorphous carbon materials which can be directly grown on silicon nitride via chemical vapor deposition,^50,51^ allowing fine adjustments of the layer conductivity through the precise control of the film thickness. The material of our choice, pyrolitic carbon (PyC), has shown an impressive absorption of ∼43% in the sub-THz range.^52^ We realized and investigated two different device implementations, with the same nominal geometrical parameters for the trampoline resonator and different absorbing layers, namely a 2/6 nm Cr/Au thick layer (Au device) and a 18 nm pyrolitic carbon thick layer (PyC device), respectively.

### Detector Read-Out

The devices were mounted on a ceramic piezoelectric stack actuator layer which was used to coherently excite the membrane motion (see Figure 1a). The AC drive voltage applied on the actuator was generated by the reference channel of a lock-in amplifier (LIA), whose input channel was connected to the TMB contact to sense the magnetomotive voltage Δ*V*, induced by the modulation of the concatenated magnetic flux due to the vibrational displacement. TMB, actuator and static magnets were mounted on a custom-made printed circuit board; this was inserted in a vacuum chamber at ∼5 × 10^–4^ mbar to reduce atmospheric viscous damping. Optical access was enabled through a cyclic-olefin-copolymer (COC) window. The vacuum chamber was additionally mounted on a planar moving stage (*xy* plane), used to scan the TMB position for extended imaging. The illumination was done via a continuous wave, 30 mW, 140 GHz source focused on the device with two parabolic mirrors (not shown in the sketch of Figure 1a).

The concept behind the detection mechanism lies in the shift of the resonant frequency of specific mechanical modes due to thermally induced device deformations, namely thermal expansion and tensile stress reduction. The temperature change is accordingly caused by the absorption of the electromagnetic radiation which one wants to detect. Sweeping the driving frequency, it is possible to directly acquire the whole mechanical spectrum and evaluate the frequency shift Δ*f* of specific features of the “bright” spectrum (*A*_b_) from the “dark” (*A*_d_) one (Spectral Scan, see Figure 1c). In our operating conditions, the frequency shift scales linearly with the illuminating intensity, as demonstrated with a similar device in a previous work,^32^ allowing the use of this operating mode with large dynamic ranged signals as well as for the direct acquisition of images, as reported, for example, in Figure 1b, where the source focused beam profile has been imaged via spatial scan. The linear response allows a direct conversion of the image from frequency shift to impinging power; here this was done by imposing the proper beam normalization to its total power, which has been independently measured through a calibrated Golay cell detector, taking also into account the absorption of the COC window, which at this frequency stands around 50%.

Note that the spectral scan is generally a slow detection method, often limited by the acquisition time of lock-in amplifiers due to the long frequency sweeps, especially when multiple devices are simultaneously investigated.

Faster detection protocols with a reduced dynamic range rely instead on operating with a single frequency driving/demodulating tone *f*_D_ and exploiting the transduction effect in an open-loop configuration, as illustrated in the transduction scheme of Figure 1d. In our device, the transduction detection speed can reach video-rate,^32^ limited by the thermal relaxation dynamics. This process, with Q-factors for our devices ranging between 10^3^ and 10^5^, fully dominates the dynamics of the entire TMB upon illumination. In the transduction scheme, the frequency shift is directly converted into a read-out voltage Δ*V*_LI_ which depends on the difference between dark and bright spectrum amplitudes at *f*_D_, *A*_d_(*f*_D_) and *A*_b_(*f*_D_), respectively. For the detection of weak signals, the bright spectrum can be recast as a frequency shift of the dark spectrum by a vanishing δ*f*, obtaining:

$$\Delta V_{\mathrm{LI}}∼A_{d}(f_{D})-A_{d}(f_{D}+\delta f)∼\frac{dA_{d}}{df}{|}_{f=f_{D}}\delta f$$ 1

and giving a read-out voltage directly proportional to the first derivative of the spectral amplitude at *f*_D_. Changing *f*_D_ allows one to explore different regions of the signal derivative, which can be very large in asymmetric and nonlinear resonances. In particular, our resonance shows both Fano interference and nonlinear hardening due to the Duffing effect, which is known to produce an increase in the steepness of the spectral features. This is illustrated in Figure 1e, which displays typical resonance lineshapes of a PyC device at different driving strengths: one can see that the already asymmetric Fano resonance becomes steeper around 525 kHz due to the Duffing effect for the larger driving voltage amplitude.

## Spectral Transduction

We evaluated the effect of asymmetric lineshapes on the NEP, one of the key parameters for detector performance. Typical amplitude spectra of the PyC device, demodulated with a sweeping mechanical driving voltage of 190 mV and with on/off source, respectively, are shown in Figure 2.

**Figure 2:**
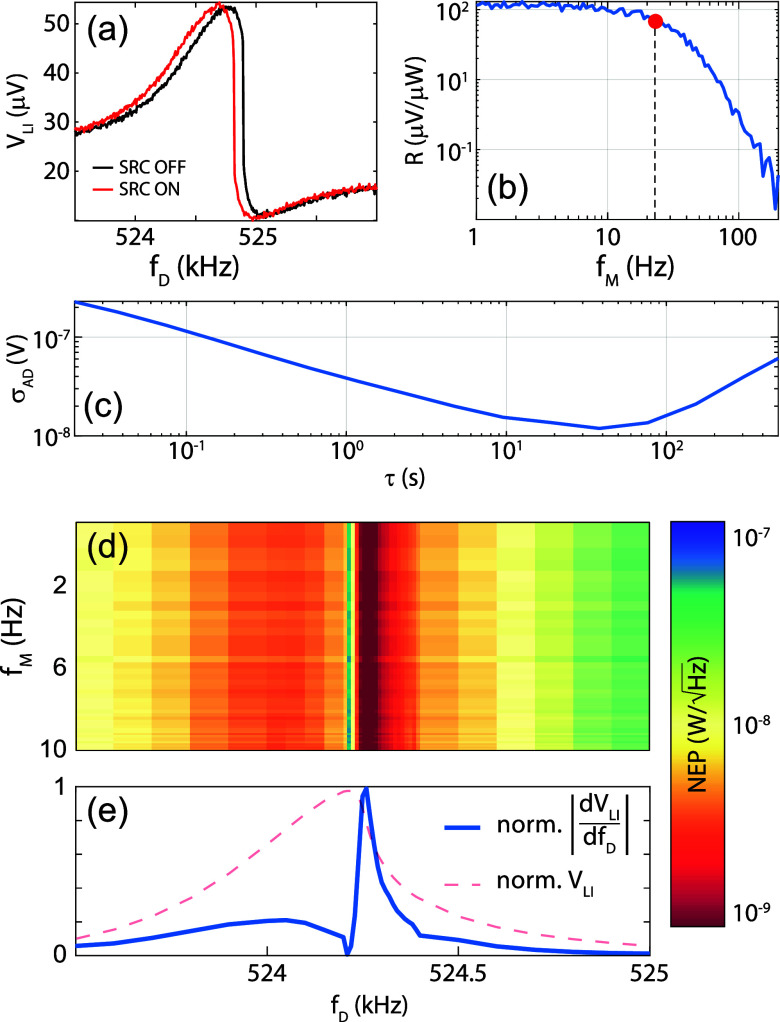
(a) Typical on/off spectra for the PyC device with a 190 mV piezo driving voltage. (b) Dynamic responsivity for the PyC device. The red dot indicates the cutoff frequency. (c) Voltage Allan deviation for the PyC device with a demodulation bandwidth of 200 Hz, evaluated at the drive frequency *f*_D_ = 524.21 kHz corresponding to the maximum amplitude of the resonant peak at 90 mV drive. (d) Spectrogram of PyC device NEP. The minimum NEP can be found around 524.26 kHz. (e) Normalized derivative and corresponding OFF spectrum (dashed) for the PyC device with a 90 mV piezo driving voltage.

Comparing dark and bright spectrum we can extract the device static frequency responsivity, using the calibrated intensity map of Figure 1b for estimating the radiation power illuminating the detector. Although one could consider the power deposited on the whole membrane area (300 × 300 μm) or the diffraction-limited area λ^2^/4 ≈ 1.15 mm^2^, for the sake of comparison we adopted the same methodology commonly used in the literature^53^ and considered the power illuminating the absorbing layer region (60 × 85 μm). This yields an illuminating power of *P*_*i*_ ∼ 0.26 μW and ∼ 0.48 μW for the PyC and Au device, respectively. Note that the different powers are due to the slightly different *xy* positions where we placed the TMBs; moreover we did not consider the absorbed power but rather the one impinging on the device surface.

Operating the device in transduction mode and considering a weak signal approximation, a static voltage responsivity can be defined for each driving frequency *f*_D_ as

$$R_{V0}(f_{D})∼\frac{dV_{\mathrm{LI}}}{df}{|}_{f=f_{D}}\left(\frac{\Delta f}{P_{i}}\right)=\frac{dV_{\mathrm{LI}}}{df}{|}_{f=f_{D}}R_{f0}$$ 2

where the static frequency responsivity *R*_*f*0_ = Δ*f*/*P*_*i*_ can be estimated from the results of Figure 2a, and represents the pure frequency shift of the resonator due to the heating from the impinging radiation. The dynamic responsivity *R* was then evaluated by combining the static voltage responsivity *R*_V0_(*f*_D_) with the Bode frequency response, obtained using a square wave modulation of the source in a frequency range *f*_M_ from 1 to 120 Hz, with an external electrical signal provided by the LIA. A typical dynamic responsivity measured at a driving frequency of 524.8 kHz is reported in Figure 2b. As expected, the thermal response of the membrane acts as a low-pass filter, with cutoff frequency given by the inverse of thermal relaxation time τ_th_. The cutoff frequency extracted from the Bode is about 20 Hz, as indicated by a red dot in Figure 2b, and corresponds to a τ_th_ ∼ 8 ms, compatible with other reports in the literature.^36,53^

Next, the device noise was measured in dark conditions, by calculating the Voltage Allan deviation σ_*AD*_, which provides a time-domain characterization of the resonator frequency stability. The Voltage Allan deviation was computed from amplitude signal recordings over 1 min at various driving frequencies, using the LIA, with demodulation bandwidth of 10 Hz, which lies below the thermal cutoff frequency of the device. A representative measurement from a longer acquisition is shown in Figure 2c, using a demodulation bandwidth of 200 Hz to show the behavior at lower τ. The typical behavior is observed: at short averaging times τ, the Voltage Allan deviation follows a ∝ τ^–1/2^ trend, indicative of white frequency noise dominance (see Supporting Information), while at longer τ, the impact of frequency drift becomes evident.

Voltage Allan deviation and dynamic responsivity can be combined to yield the Noise-Equivalent Power; considering the time-dependence of both the Bode plot of Figure 2b and the noise contribution of Figure 2c, as expected, the NEP is a function of the frequency of modulation of the incoming signal (*f*_M_). Moreover, given the strong asymmetry of our lineshapes, in our device the NEP is also function of the mechanical driving frequency *f*_D_:

$$\mathrm{NEP}(f_{M},f_{D})=\frac{\sigma_{\mathrm{AD}}\sqrt{2\tau }}{R}$$ 3

The full NEP spectrogram for the PyC device excited with a 90 mV driving tone can then be expressed in the *f*_M_ – *f*_D_ plane, as reported in Figure 2d. It is significant to observe that there is a drastic reduction of the NEP at a driving frequency of *f*_D_ = 524.26 kHz, reaching values below $1\;\mathrm{nW}/\sqrt{\mathrm{Hz}}$ , significantly reduced with respect to the rest of the spectrum. Unsurprisingly, this driving frequency corresponds to the spectral region with the highest slope of the mechanical signal, as can be seen from Figure 2e, where we reported the normalized modulus of the derivative calculated from the resonance line shape (also reported as a dashed line).

Qualitatively similar effects are present at different driving voltages: higher drives lead to even sharper lineshapes, with a corresponding decrease in the NEP in narrow spectral regions. Conversely, lower drives produce an increase in the NEP, albeit showing the best performances in a broader spectral range. This concept is directly linked to the device dynamic range of operation: large spectral regions with significant slopes are most beneficial to the use of the detector for imaging or as a power meter; at the other limit, extremely large slope in very narrow frequency ranges, we expect the device to operate as a threshold-switching detector, better suited for the recognition of single events (i.e., laser pulses), as will be discussed later.

As we have illustrated, a critical parameter in our operating scheme is the derivative of the mechanical resonance: changing the transduction frequency can dramatically change the device response. For example, Figure 3a shows the normalized mechanical spectrum of PyC device under a driving voltage of 112 mV.

**Figure 3:**
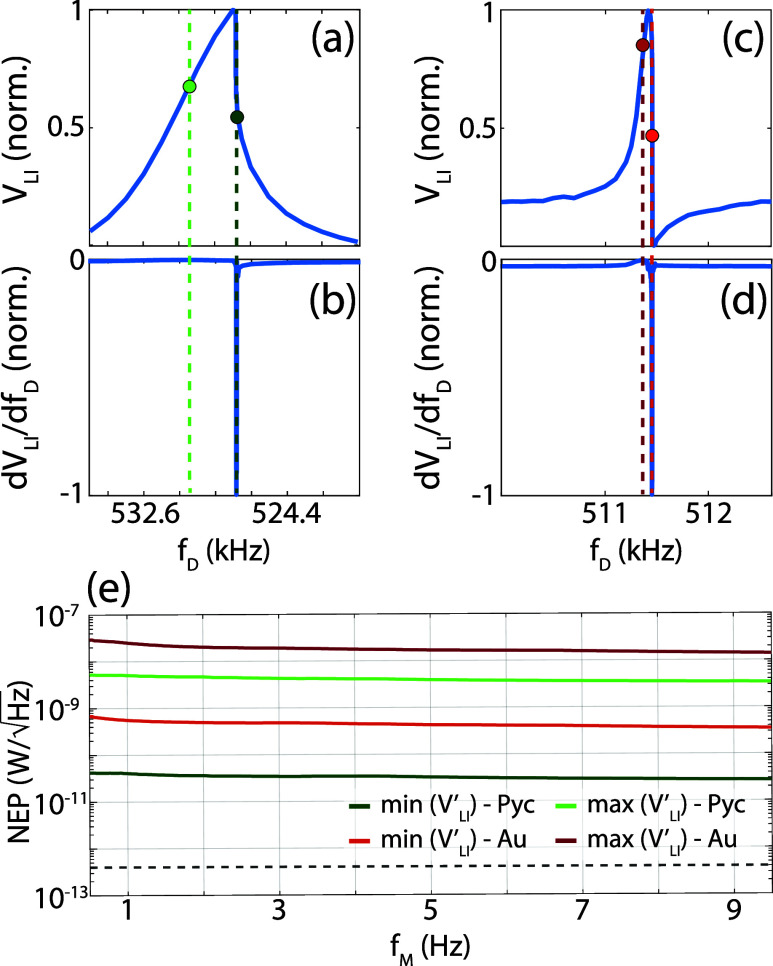
Normalized vibrational spectrum (a) and its first derivative (b) for the PyC device. Normalized vibrational spectrum (c) and its first derivative (d) for the Au device. The dashed lines indicate in both cases the position of the maximum and minimum derivative, respectively. (e) NEP evaluated for *f*_D_ corresponding to the maximum (i.e., most positive) and minimum (i.e., most negative) of the derivative for both devices. The dashed line indicates the theoretical thermal limit of the NEP, calculated for the PyC device.

The asymmetry becomes even more manifest by looking at the derivative: the largest negative slope is about 5.6 times the largest positive one, see the green dots and lines in Figure 3b. The Au device shows a qualitatively similar asymmetry, as shown in Figure 3c,d. The two devices are nominally identical apart from the different absorption layer. Differences in their resonant spectra can be ascribed to the different experimental assembly, starting from the local properties of the piezoelectric actuator where they are glued upon. The piezoelectric stack represents a cross-talk channel strongly impacting the Fano factor, see a more detailed modeling in ref 36. For a fair device comparison, we then evaluated the driving voltage leading to the bifurcation point (discussed in more detail later): this acts as a common reference, since we expect both devices to have the same vibrational amplitude at bifurcation. Scaling from this value, we applied a driving voltage of 118 mV to the Au device (Figure 3c,d), so that it can be compared to the 112 mV drive of the PyC one (Figure 3a,b). The Au device also exhibits a strong asymmetry (roughly a 37.5 times increase of the negative to the positive slope) and generally a comparable but smaller derivative with respect to the PyC device.

The NEP evaluated by setting *f*_D_ at the maximum and minimum slopes of both devices is reported in Figure 3e as a function of the modulation frequency of the 140 GHz source. All NEP curves remain relatively constant as a function of *f*_M_, consistent with the results of Figure 2d, and show an overall low value which strongly depends of the chosen transduction frequency. As expected from eqs 3 and 2, the NEP is inversely proportional to the derivative, resulting in net reductions of about a factor of 117 and 37.5 when driving the two devices at their largest positive slopes or at their largest negative ones, in good agreement with what one would expect from the results of Figure 3b,d. Note that the NEP reductions are obtained within the same mechanical device, and obtained just by changing *f*_D_. Moreover, the PyC device shows about an order of magnitude improvement with respect to the Au device which originates from the different absorbance of the two materials at 140 GHz. Interestingly, comparing the best NEP values in the two devices, appropriately rescaled to account for the different line shape slopes, gives an absorption-induced enhancement of ∼7.94. This is in good agreement with the ratio of the static frequency responsivities, which are independent of the resonator line shape and therefore proportional to the material absorption. We obtain *R*_*f*0_^PyC^/*R*_*f*0_^Au^ = 6.85, with *R*_*f*0_^PyC^ ∼ 497 MHz/W and *R*_*f*0_^Au^ ∼ 72.6 MHz/W.

From this observation, we can estimate the absorbance of the metallic layer, starting from an experimentally measured PyC film absorbance of ∼40% in the far-infrared range.^52^ The obtained Cr/Au absorbance of ∼10% is compatible with multilayer simulations using the experimentally estimated refractive indices of Si_3_N_4_ and metals,^54^ which returns a numerical value of 4 ± 0.5% for a continuous film, whereas we can assume a further enhancement due to the layer inhomogeneity. For comparison with other IR/THz MEMS-based detection techniques, we also report the relative responsivity, defined as $R_{r}=\frac{\Delta f}{f_{0}P_{i}}$ , for both devices. We obtain *R*_r_^PyC^ ≃ 947.1 W^–1^, *R*_r_^Au^ ≃ 141.8 W^–1^, where *f*_0_ is the resonance frequency corresponding to the maximum amplitude of the resonant peak used in the measurement of Δ*f*.

The best NEP we obtained was about 30 $\mathrm{pW}/\sqrt{\mathrm{Hz}}$ for the PyC device. As reported in Figure 3e, we still have room toward the fundamental NEP limit for a detector operating at a temperature *T*, set by thermal fluctuations in the system and defined as^55^

$${\mathrm{NEP}}^{*}=\sqrt{16k_{B}T^{5}\sigma A}$$ 4

where *k*_B_ is the Boltzmann constant, σ is the Stefan–Boltzmann constant and A is the absorber area. Even if some thermal detectors shows exceptional performances and metrics close to the thermal limit in the mid-infrared range, they rely on all-optical probes, which are known to inject less noise to the system with respect to the all-electrical ones employed here.^53^ Conversely, all-electrical read-out is better suitable for portability and integration and our NEP compares well with some of the best commercial devices in the sub-THz range, which have NEPs around 10 $\mathrm{pW}/\sqrt{\mathrm{Hz}}$ .^56^ A detailed comparison of key metrics of emerging MEMS-based detectors is reported in Table 1, where one can compare different readout methods (electrical/optical), frequency range of operation, thermal relaxation time, responsivity and NEP. As can be seen, the bolometers characterized here are well positioned among similar systems operating within similar frequency range.

**Table 1:** Comparison of MEMS-Based Detectors Operating in the Infrared Spectral Range^a^

geometry	readout	frequency range	τ_th_ (ms)	responsivity	NEP $\left(\mathrm{pW}/\sqrt{\mathrm{Hz}}\right)$
trampoline^32^	O	140 GHz	25		100
square membrane^53^	O	15–210 THz	14		27
square membrane^34^	O	0.5–3 THz	200	120 W^–1^	36
cantilever^57^	O	3.24–3.98 THz	100	24.8 μm/μW	38.2
cantilever (meta-atom)^58^	O	2.6 THz	0.003		16,000
this work	E	140 GHz	8	947.1 W^–1^	30
trampoline^33^	E	12–300 THz	4	11000 W^–1^	7
beam^59^	E	1–10 THz	0.88	149 W^–1^	36
drum^60^	E	6–60 THz	17	343 W^–1^	320

a Key metrics are Geometry, Readout (O-optical/E-electrical), Frequency Range of operation, thermal relaxation τ_*th*_, Responsivity and NEP.

The main drawback of our technique lies in the reduced bandwidth where we find very large derivatives. This directly translates into a reduction of the detector dynamic range (i.e., the ratio between the maximum and minimum detectable power). As a quantitative example, with the driving condition of Figure 3, the PyC device negative derivative peak has a line width of about 50 Hz, limiting its use to weak signals with power less than roughly 100 nW, as estimated by considering the static frequency device responsivity. Nevertheless, this is still a useful range given the interest, for example, for the detection of passive blackbody in the THz range. Furthermore, the detector can also operate with stronger signals by transducing in different spectral regions or at reduced driving strengths, albeit at the expense of responsivity. As an alternative approach, it is possible to compare the full spectrum in bright and dark conditions; this preserves a high responsivity while extending the dynamic range, at the price of greatly reduced operational speed. A characterization of the linear response regime for a weak drive (90 mV) of the PyC device has been reported in the Supporting Information. Spanning the average illuminating power via fast TTL modulation of the 140 GHz source, we show a linear scaling of both the frequency shift and single-frequency readout voltages in an experimentally accessible dynamic range of tens of nW.

A further increase of the driving voltage leads the system to highly nonlinear regimes which, in the case of Duffing nonlinearities, can eventually reach multistable or chaotic motion.^40^ As an example, Figure 4a,b reports the comparison of an asymmetric, single-solution resonance (red curve) and a regime past the bifurcation point (blue curve) for both devices under investigation. The multistable regime is characterized by abrupt jumps resulting from switching between two stable solutions; the onset of multistability essentially depends on the vibrational amplitude, since the Duffing nonlinearity enters the equation of motion with a cubic displacement term.^36^ The bifurcation point from a single to multiple dynamical solutions has been taken as a common reference to compare both devices.

**Figure 4:**
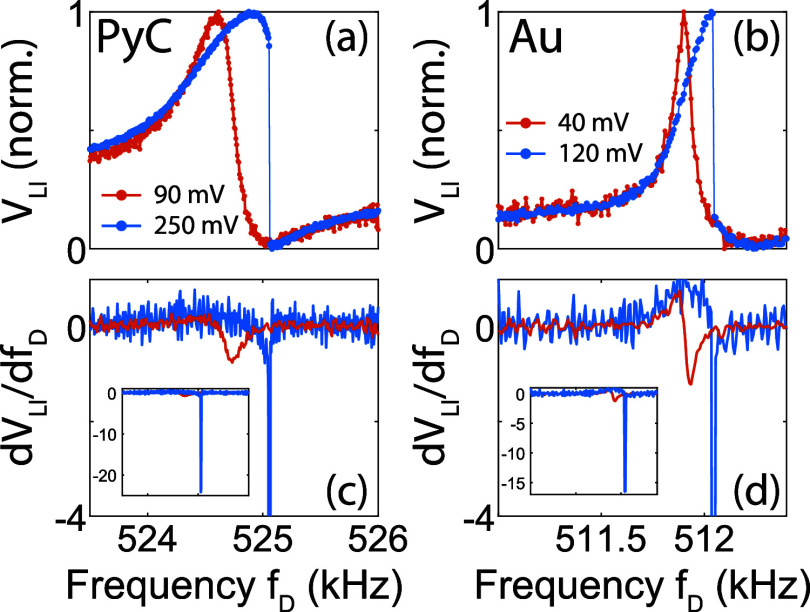
Normalized vibrational spectrum (a) of the PyC device under a weak (90 mV) and a strong (250 mV) driving voltage amplitude, respectively. The large drive amplitude leads to a multistable regime where the oscillator jumps from one stable solution to the other. The multistability leads to delta-like responsivity, as seen in the zoomed-in calculated first derivative of (c) (inset: full scale derivative comparison). (b, d) Similar measurements for the Au device.

One can think of the switching as a “line with infinite slope”, which looks appealing for transduction detection. Unfortunately, in this case the derivative tends to a δ-function, as can be seen from the normalized numerical derivates reported in Figure 4c,d, respectively. Evaluating the figures (and the insets for the full range of the *y* axis), one can expect an extreme enhancement of device performance in terms of responsivity, which comes along with a vanishing dynamic range.

Since diverging derivatives can be found at a single frequency point, this particular regime of operation does not allow utilizing the device as an intensity detector; conversely it looks promising for a threshold-based sensor, where the discontinuity in the read-out can be triggered by specific events over a certain limit (i.e., single photons, light pulse detection, temperature change, mass loading). In a scheme similar to the superconducting optical detectors^20^ or bifurcation-based sensors^25–29^ in other nonlinear systems, this operating regime adds to the potential and possibilities lying in thermomechanical bolometer devices.

## Conclusions

While increasing the quality factor in resonant detectors generally enhances device performance, consistently achieving high values can be challenging, especially in the context of mass production for real-world applications. In this work, we presented an alternative approach to locally achieve high responsivity and low NEP in transduction detection experiments by exploiting device nonlinearities. Through careful characterization of thermomechanical bolometers, we demonstrated that the device NEP under 140 GHz illumination is strongly dependent on the driving strength and transduction frequencies. In the optimal operating range, we achieved a NEP of 30 $\mathrm{pW}/\sqrt{\mathrm{Hz}}$ for a TMB employing pyrolitic carbon as an absorbing layer, which exhibits a 40% absorbance in the sub-THz range under investigation.^52^ Additionally, by further increasing the driving strength and entering a highly nonlinear regime, we propose leveraging the same platform for threshold signal detection. In this regime, the system can transition between stable solutions in response to external perturbations of sufficient magnitude, with a behavior similar to superconducting or bifurcation-based detectors, opening up further possibilities for our technology.

While our TMBs, if operated as high-dynamic range detectors, do not achieve a NEP as low as other uncooled thermomechanical systems (which are rapidly approaching the fundamental detection limit essentially thanks to all-optical probing^53^), the devices investigated here offer superior integrability and large-scale processing. This has already been demonstrated in realized 30-pixel array detectors with massively multiplexed readout,^36^ and more recently scaled up to 256 pixels.^61^ Moreover, individual control of the TMB within the array enables nonlinearity enhanced detection, leading to an overall improvement in device performance, although challenges remain in ensuring uniform pixel characteristics, including both linear and nonlinear behavior of the mechanical resonators. The reduced dynamic range still allows for numerous application opportunities, including the selective detection of the THz portion of blackbody radiation, which could enable source-free THz spectroscopy. Although our experimental demonstration focused on sub-THz TMBs, the approach we employed can be broadly extended to a wide class of detectors operating in a transduction scheme, where overall device responsivity can be improved with only minimal modifications to the structure.

## Supplementary Material



## Notes

Derivation of the equation used to estimate the noise-equivalent power, including all relevant steps and assumptions; estimate of the overall thermal dissipation of the devices; and characterization of the linear response regime of the PyC device (PDF)
