# Supplementary material for: Enhanced Sensitivity of Sub-THz Thermomechanical Bolometers Exploiting Vibrational Nonlinearity
Source: ACS Photonics. 2025 Dec 26;13(1):58–66. doi: 10.1021/acsphotonics.5c01613 (PMC12784404; doi:10.1021/acsphotonics.5c01613)
Supplement: Supplementary file 1 [file ph5c01613_si_001.pdf]

Supplementary Information for:
Enhanced sensitivity of sub-THz thermomechanical bolometers exploiting vibrational nonlinearity

L. Alborghetti,[1] B. Bertoni,[1,2,*] L. Vicarelli,[1] S. Zanotto,[2] S. Roddaro,[1,2] A. Tredicucci,[1,2] M. Cautero,[3,4] L. Gregorat,[5,4] G. Cautero,[4] M. Cojocari,[6] G. Fedorov,[6] P. Kuzhir,[6] and A. Pitanti[2,1,†]

[1]*Department of Physics, University of Pisa, Largo B. Pontecorvo 3, 56127 Pisa - Italy*
[2]*NEST, CNR Istituto Nanoscienze, piazza San Silvestro 12, 56127 Pisa - Italy*
[3]*Department of Physics, Università degli Studi di Trieste, Piazzale Europa 1, 34127 Trieste, Italy*
[4]*Elettra - Sincrotrone Trieste S.C.p.A., Strada Statale 14, km 163.5, 34149 Trieste, Italy*
[5]*Department of Engineering and Architecture, Università degli Studi di Trieste, Via Alfonso Valerio 6/1, 34127 Trieste, Italy*
[6]*Department of Physics and Mathematics, Center of Photonics Sciences, University of Eastern Finland, Yliopistokatu 7, FI-80101 Joensuu, Finland*

Number of pages: [5]
Number of figures: [1]
Number of tables: [0]

* benedetta.bertoni@phd.unipi.it
† alessandro.pitanti@nano.cnr.it

NEP DERIVATION

In this Supplementary Information section, we detail the derivation of the Noise-Equivalent Power (NEP) used in the main text, focusing specifically on the regime dominated by white frequency noise. As shown in the Allan deviation measurements (see Figure 2(c) in the main paper), this is the relevant noise contribution in the range of averaging times we investigate.
The Allan variance is a widely used tool to characterize frequency fluctuations over time, but in principle it can also be applied to characterize general signal fluctuations[1–3], such as the amplitude of our signal as in the main text. It is related to the power spectral density (PSD) of the fluctuations, $S_y(f)$, by the following integral:

$$\sigma^2_{AD}(\tau) = 2\int_0^\infty S_y(f)\cdot\frac{\sin^4(\pi f\tau)}{(\pi f\tau)^2}\,df. \tag{1}$$

where τ is the averaging time and y is the measured variable whose noise we want to characterize (for example, the readout voltage amplitude in the devices reported in the main text). The transfer function in the integrand,

$$H(f) = \frac{\sin^4(\pi f\tau)}{(\pi f\tau)^2} \tag{2}$$

peaks at $f \approx \frac{1}{2\tau}$, meaning the Allan variance primarily captures fluctuations at this characteristic frequency.
In the case of white frequency modulation (white FM), the PSD is flat[4]:

$$S_y(f) = h_0 = \text{constant}. \tag{3}$$

Substituting into the Allan variance expression, we get:

$$\sigma^2_{AD}(\tau) = 2h_0\int_0^\infty \frac{\sin^4(\pi f\tau)}{(\pi f\tau)^2}\,df \tag{4}$$

To evaluate this integral, we use the change of variable: $x = \pi f\tau \Rightarrow df = \frac{dx}{\pi\tau}$
Thus, the integral becomes:

$$\int_0^\infty \frac{\sin^4(\pi f\tau)}{(\pi f\tau)^2}\,df = \frac{1}{\pi\tau}\int_0^\infty \frac{\sin^4(x)}{x^2}\,dx. \tag{5}$$

From standard integral tables, we know:

$$\int_0^\infty \frac{\sin^4(x)}{x^2}\,dx = \frac{\pi}{4}. \tag{6}$$

so the full integral evaluates to:

$$\int_0^\infty \frac{\sin^4(\pi f\tau)}{(\pi f\tau)^2}\,df = \frac{1}{4\tau}. \tag{7}$$

Finally, we obtain the Allan variance under white FM noise:

$$\sigma^2_{AD}(\tau) = 2h_0\cdot\frac{1}{4\tau} = \frac{h_0}{2\tau}. \tag{8}$$

RELATION BETWEEN NEP AND $S_y(f)$

The NEP is related to the power spectral density $S_y(f)$ and the responsivity R by:

$$\mathrm{NEP} = \frac{\sqrt{S_y(f)}}{R}$$

Under the white noise assumption, and considering the correspondence between the peak of the Allan variance filter and $f \approx \frac{1}{2\tau}$, we can express $S_y(f)$ in terms of the Allan deviation:

$$S_y(f) = 2\tau\sigma_{AD}^2(\tau)$$

which leads to:

$$\mathrm{NEP} = \frac{\sigma_{\mathrm{AD}}(\tau)\sqrt{2\tau}}{R}.$$

CONTRIBUTION OF THERMAL RADIATION

In the main text, we stated that the transduction detection speed is limited by thermal conduction through the tethers of the detector. To support this statement, in this section of the Supporting Information we estimate the overall thermal dissipation, taking into account both thermal conduction and thermal radiation.
The total thermal conductance in our system can be written as a sum of heat transport in the solid region (through the tethers towards the substrate) and of radiative transport, valid for small changes in temperature around $T_0 \sim 300$ K[5].

$$G = G_{cond} + G_{rad} = \frac{8}{L_t}(w_t h k_{Si_3N_4} + w_c h_c k_{Au}) + 4((8wL_t + 2(A_{pad} - A_{abs}))\epsilon_{Si_3N_4} + 2A_{abs}\epsilon_{abs})\sigma_{SB}T_0^3 \quad (9)$$

Where we have contributions from the trampoline geometry:
$h = 300$ nm membrane thickness, $h_c = 50$ nm contacts thickness, $w_t = 12$ μm tether width, $w_c = 10$ μm contacts width, $L_t = 100\sqrt{2}$ μm tether length, $A_{pad} = 10^4$ μm^2 central pad area, $A_{abs} = 60$ μm $\times$ 85 μm absorber area
and material composition:
$\epsilon_{Si_3N_4}$ silicon nitride emissivity ($\sim$ 0.2 for our thickness[6]), ϵ_{abs} absorber emissivity ($\sim$ 0.4 for PyC), $k_{Si_3N_4} \approx 3$ W m^{-1}K^{-1} silicon nitride thermal conductivity and $k_{Au} \approx 317$ W m^{-1}K^{-1} thermal conductivity of bulk gold (thus likely an overestimate for thin films). $\sigma_{\mathrm{SB}} = 5.670 \times 10^{-8}$ W m^{-2}K^{-4} is the Stefan-Boltzmann constant. Substituting the numbers, we obtain:

$$G_{\mathrm{cond}} = \frac{8}{L_t}(w_t h k_{Si_3N_4} + w_c h_c k_{Au}) \approx 9.57 \times 10^{-6} \text{ W/K} \quad (10)$$

$$G_{\mathrm{rad}} \approx 5.36 \times 10^{-8} \text{ W/K} \quad (11)$$

where we considered $\epsilon_{abs} \sim 0.4$ for the PyC device.
Thus, thermal conduction through the tethers dominates, with:

$$\frac{G_{\mathrm{cond}}}{G_{\mathrm{rad}}} \approx 178.5 \quad (12)$$

A similar estimate can be carried out for the Au absorber. However, since this is not a uniform film, it is difficult to estimate an accurate value for ϵ_{abs}. Considering a very strong overestimation, assuming a thin, uniform gold film with impedance matching leading to 50% absorption[7], we have $\epsilon_{abs} \sim 0.5$. Even under this condition, the radiative thermal conductance is $G_{\mathrm{rad}} \approx 5.99 \times 10^{-8}$ W/K which remains two orders of magnitude smaller than G_{cond}.

POWER MEASUREMENTS

The power calibration of the 140 GHz source, obtained by integrating its spatial map, is valid under the assumption of linearity in the response of the detector. We report here a power characterization which showed the linear proportionality of mechanical frequency shift and illuminating power. A variable power was obtained by imposing a time-dependent TTL modulation on the 140 GHz source. Using an arbitrary waveform generator, we generated a square wave at 200 Hz with variable duty-cycle. Being the modulation frequency faster than the inverse of thermal relaxation time as well as slow regarding the mechanical vibrations, we can consider the average power illuminating the device directly proportional to the fractional duty-cycle. We started by considering the **PyC device** with a weak actuation drive of 90 mV, producing the lineshapes reported in Fig. 1 (a). At low drive, the Duffing effect is negligible and the experimental data can be fitted using a Fano function[8]:

$$f(\Omega) = A + [4\sin(\delta)]^2 \frac{\cot(\delta) + \Omega}{1 + \Omega^2} \tag{13}$$

where Ω is a function of the frequency ν, $\Omega = 2(\nu - \nu_0)/\gamma$, with ν_0 and γ frequency and linewidth of the resonance and δ is the dephasing between the resonance and continuum. The best fit with Eq. (13) has been reported as a continuous line in Fig. 1 (a). Afterwards the TMB was illuminated at different duty-cycles, from 22% to 100 %, and the resulting lineshapes fitted with Eq. (13). Figure 1 (b) reports the extracted ν_0 as a function of the average power illuminating the absorber surface, as done in the calibration procedures in the main text. Every measurements have been repeated several times, producing the errorbars plotted in the figure. The frequency shifts scales linearly with the power; from the slope of the curve we obtain a static frequency responsivity of $R_{\nu_0} = 490$ MHz/W, which well compares with the value estimated in the main text. While the linearity of the frequency response is well observed for a wide power ranges, as reported in other publications[9], the reduced dynamic range here explored grants also a linear voltage response. As an example, we have reported in Fig. 1 (c) the voltage shift under illumination at single transduction frequencies depicted as dashed lines in panel (a). As expected, the transducing frequency close to a minimum shows almost zero signal for all the powers (red data), while the transducing frequencies in regions with higher slopes produce signals proportional to the slope itself (blue and black data). Interestingly all voltage shift scale linearly with illuminating powers, demonstrating the linear detector response in the tens of nW power range.

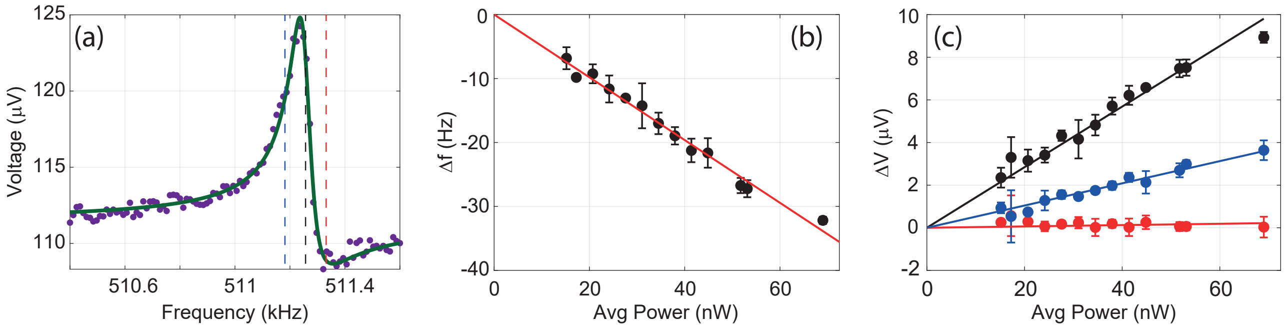

FIG. 1. (a): mechanical resonance at source off, the green line is the best fit with a Fano function. (b): linear frequency shift of the resonance under different illuminating powers. The continuous line is the linear best fit. (c): linear voltage shift under different illuminating powers for fixed transduction frequencies. The frequencies selected are indicated with dashed lines in panel (a), the continuous lines are linear best fits.

[1] F. Tricot, D.-H. Phung, M. Lours, S. Guérandel, and E. De Clercq, Power stabilization of a diode laser with an acousto-optic modulator, Review of Scientific Instruments **89**, 113112 (2018).

[2] T. Witt, Using the allan variance and power spectral density to characterize dc nanovoltmeters, Instrumentation and Measurement, IEEE Transactions on **50**, 445 (2001).

[3] T. J. Witt and D. Reymann, Using power spectra and allan variances to characterise the noise of zener-diode voltage standards, IEE Proceedings - Science, Measurement and Technology **147**, 177 (2000).

[4] E. Rubiola, Phase noise and frequency stability in oscillators, in *Microwave and Wireless Synthesizers*, edited by U. L. Rohde, E. Rubiola, and J. C. Whitaker (Wiley, 2021) 2nd ed., Chap. 2.

[5] K. Kanellopulos, F. Ladinig, S. Emminger, P. Martini, R. G. West, and S. Schmid, Comparative analysis of nanomechanical resonators: sensitivity, response time, and practical considerations in photothermal sensing, Microsystems & Nanoengineering **11**, 10.1038/s41378-025-00879-6 (2025).

[6] C. Zhang, M. Giroux, T. A. Nour, and R. St-Gelais, Radiative heat transfer in freestanding silicon nitride membranes, Phys. Rev. Appl. **14**, 024072 (2020).

[7] N. Luhmann, D. Høj, M. Piller, H. Kähler, M.-H. Chien, R. G. West, U. L. Andersen, and S. Schmid, Ultrathin 2 nm gold as impedance-matched absorber for infrared light, Nat Commun **11**, 2161 (2020).

[8] M. F. Limonov, M. V. Rybin, A. N. Poddubny, and Y. S. Kivshar, Fano resonances in photonics, Nat. Photonics **11**, 543 (2017).

[9] L. Vicarelli, A. Tredicucci, and A. Pitanti, Micromechanical Bolometers for Subterahertz Detection at Room Temperature, ACS Photonics **9**, 360 (2022).
